# Improved Visualization of Hydroacoustic Plumes Using the Split-Beam Aperture Coherence

**DOI:** 10.3390/s18072033

**Published:** 2018-06-25

**Authors:** Ann E. A. Blomberg, Thomas C. Weber, Andreas Austeng

**Affiliations:** 1 Digital Signal Processing and Image Analysis Group, Department of Informatics, University of Oslo, P.O. Box 1080 Blindern, 0316 Oslo, Norway; andrea@ifi.uio.no; 2 Center for Coastal & Ocean Mapping/Joint Hydrographic Center, University of New Hampshire, Durham, NH 03824, USA; weber@ccom.unh.edu

**Keywords:** marine gas seeps, gas seep detection, coherence, coherence factor, broadband split beam echo sounder, physical oceanography, scattering layers, hydroacoustic plumes

## Abstract

Natural seepage of methane into the oceans is considerable, and plays a role in the global carbon cycle. Estimating the amount of this greenhouse gas entering the water column is important in order to understand their environmental impact. In addition, leakage from man-made structures such as gas pipelines may have environmental and economical consequences and should be promptly detected. Split beam echo sounders (SBES) detect hydroacoustic plumes due to the significant contrast in acoustic impedance between water and free gas. SBES are also powerful tools for plume characterization, with the ability to provide absolute acoustic measurements, estimate bubble trajectories, and capture the frequency dependent response of bubbles. However, under challenging conditions such as deep water and considerable background noise, it can be difficult to detect the presence of gas seepage from the acoustic imagery alone. The spatial coherence of the wavefield measured across the split beam sectors, quantified by the coherence factor (CF), is a computationally simple, easily available quantity which complements the acoustic imagery and may ease the ability to automatically or visually detect bubbles in the water column. We demonstrate the benefits of CF processing using SBES data from the Hudson Canyon, acquired using the Simrad EK80 SBES. We observe that hydroacoustic plumes appear more clearly defined and are easier to detect in the CF imagery than in the acoustic backscatter images.

## 1. Introduction

Detecting marine gas seeps using hydroacoustics started already in 1984, with many studies since then aiming at mapping and monitoring marine seep sites [1,2,3,4,5]. Natural seepage of methane is common in many regions where geological structures such as pockmarks, mud-volcanoes, faults or salt domes allow this greenhouse gas to penetrate the seafloor and enter the water column [6]. Also, rising seawater temperatures may trigger the release of large amounts of gas currently trapped either in permafrost, or as gas hydrates stabilized by high pressure and low temperatures [7,8,9,10]. In addition to naturally occurring seepage, leakage from offshore infrastructure such as pipelines and wells represents an environmental as well as an economical risk factor.

Gas bubbles in water are visible in acoustic backscatter images (sonar images and echograms) due to the significant contrast in acoustic impedance between gas-filled bubbles and water [11,12,13,14,15,16]. Marine gas seeps have previously been studied using a range of acoustic technologies including single- and multibeam echo sounders (MBES) [15,17,18,19,20,21,22], split-beam echo sounders (SBES) [2,7,23,24,25,26,27,28,29,30,31], sidescan sonar [32,33], and passive sonar [34,35,36]. MBES are routinely used for seafloor mapping, and are currently the prevailing method also for mapping marine gas seeps due to their high area coverage rate. Potential seep sites are usually identified in the multibeam imagery by manual interpretation of the water column data, where rising bubbles appear as strong scatterers. Rising bubbles, or hydroacoustic plumes, are often recognized as a characteristic flare shape in the acoustic imagery. Standard MBES are currently not calibrated, which limits their ability to estimate bubble flux from the measured echo intensity.

SBES have a limited field of view in the cross-track direction due to the narrow single transmit beam, but display other properties which complement MBES and have proven helpful for seep investigation and characterization. For example, recent studies show how split-beam processing using the phase difference between half-beams may be used to gain a better understanding of marine gas seeps and the evolution of bubbles as they rise through the water column [27,37]. Further, the multi-frequency or broadband capabilities of modern calibrated split-beam echo sounders can be used to characterize and potentially quantify the amount of gas escaping seep sites, by analyzing the characteristic frequency response of bubbles in water [27,38]. Since the range resolution is inversely proportional to the bandwidth, the broadband capabilities also improve the ability to investigate small-scale features in the water column such as single bubbles, fish, and horizontal scattering layers. A remaining issue is the time consuming and labor intensive process of manually identifying flares in the acoustic imagery. While progress has been made in order to automatically recognize flares [39,40], this remains challenging in cases where these are not clearly defined relative to the background.

Here, we demonstrate how the spatial coherence of the wavefield measured across the split beam sectors can be used to enhance flares in the acoustic imagery. The spatial coherence, here quantified by the coherence factor (CF), is generally high at the location of a flare while the random nature of the surrounding water column results in a low spatial coherence. The CF represents a computationally simple and easily available quantity which complements the backscatter images and may improve seep detection, especially in challenging conditions such as deep waters, small seeps, and high background noise levels. CF processing may ease the burden of manually screening large amounts of data, and also improve the detection capability of automatic algorithms by offering a better starting point. We also observe that other oceanographic features including horizontal scattering layers and single targets are more clearly defined through CF processing.

The data acquisition and initial processing required to produce acoustic backscatter and CF images is described in Section 2.1 and Section 2.2. In Section 2.3 we give a brief overview of the acoustic properties of gass-filled bubbles in water. In Section 3, we present acoustic and CF imagery from the Hudson Canyon, acquired using a Simrad EK80 SBES (Kongsberg Maritime AS, Horten, Norway) with broadband capabilities (25 to 50 kHz). MBES data acquired using the Kongsberg EM302 (Kongsberg Maritime AS, Horten, Norway) is presented to verify and compare observations. We discuss our findings and the potential usefulness of coherence processing in Section 4, and offer our conclusions from this study in Section 5.

## 2. Materials and Methods

### 2.1. Data Acquisition and Initial Processing

SBES data were acquired during a survey in the Hudson Canyon in July 2014, using a Simrad EK80 SBES mounted on the research vessel Endeavor [41] in an area of known gas seepage [42]. The SBES data used in this manuscript are the same used in [41], which also found elevated levels of aqueous CH4 at depths corresponding to the acoustic anomalies described in the current study. In [41], the authors also found acoustic anomalies in sub-bottom profiler data indicating the presence of gas below the seabed. The EK80 was equipped with a three-element wide band transducer (WBT) with a center frequency of 38 kHz and approximately 25 kHz bandwidth.

The raw EK80 data was recorded for each of the three split beam sectors, and post processed using a MATLAB processing package provided by the manufacturer, Kongsberg Maritime. This initial processing includes matched filtering using an ideal replica of the transmitted signal, as well as initial filtering and time varying gain (TVG) compensation to account for propagation losses. After initial processing, the complex-valued measurements from each of the three split beam sectors are coherently combined to form the echogram and the coherence image as described in Section 2.2.

### 2.2. Aperture Coherence and the Coherence Factor (CF)

The use of coherence in underwater acoustics is extensive and well documented [43,44]. In radar and sonar interferometry, the *interferometric* coherence refers to the degree of coherence between complex-valued images obtained simultaneously at different spatial locations, and is used as a data quality measure [45,46,47]. In [33], the interferometric coherence is used as a feature for automatic gas seep detection. *Temporal* coherence is a measure of the similarity of measurements acquired at the same spatial location but acquired with a time delay, and is useful for instance in change detection [48]. In [49], the authors use the degree of *spectral* coherence of the backscattered wavefield as a feature to differentiate between clutter and man-made objects on the seafloor or buried in the sediments.

In this work we consider the *aperture coherence*, i.e., the degree of coherence of the wavefield measured across the elements of the receiver array at a single time sample. The aperture coherence is quantified by the coherence factor (CF), defined as the ratio of the coherent sum across the array to the noncoherent sum. The CF weights are computed for each image pixel (ping *p* and range sample *r*), based on the received data samples (y[p,r]):

(1) $$\mathrm{CF}[p,r]=\frac{|\sum_{m=1}^{M}y_{m}{\left[p,r\right]|}^{2}}{M\sum_{m=1}^{M}{\left|y_{m}\left[p,r\right]\right|}^{2}}=\frac{{\left|I\right|}^{2}}{M\sum_{m=1}^{M}{\left|y_{m}\left[p,r\right]\right|}^{2}},$$

where $y_{m}\left[p,r\right]$ denotes the received complex-valued sample at element *m*. A split-beam transceiver array normally consists of three or four sectors, i.e., M=3 or M=4.

The right hand side of (1) states that the CF is proportional to ${\left|I\right|}^{2}$, where *I* is the complex-valued pixel intensity in the calibrated echogram, normalized by the noncoherently combined contributions from the split-beam sectors. Note that when computing the standard echogram, the only additional processing step required to obtain the CF weights is this local, pixel-wise normalization.

The CF can be understood in the light of the Cittert-Zernike theorem developed for the field of optics and generalized to pulse echo measurements in [50]. When there are many random scatterers within a resolution cell, the scattered wavefield will be noncoherently scattered (random phase). In this case the aperture coherence will approach the bias level (zero for an infinitely long array). For a coherent scatterer such as a fish or a single bubble and limited noise, the aperture coherence is high. In Section 3 we demonstrate that the coherence is also relatively higher than the background when a seep occupies a fraction of the acoustic footprint.

The CF was proposed by Hollman et al. as a means of quantifying the quality of each pixel in a medical ultrasound image [51]. It is known in the seismic community as semblance, and routinely used to estimate propagation velocity profiles. It has also been applied to sidescan sonar imagery for improved image contrast [52].

In some cases the CF image is computed through pixel-wise multiplication of the complex-valued backscatter image by the CF weights. This is useful for reducing noise in acoustic imagery, including random background noise and sidelobe effects. We consider the CF weights themselves a more useful tool for feature extraction. In this paper, the term CF or CF image refers to the CF weights from (1). In Section 3, we present the acoustic water column and seafloor backscatter images (*I*), i.e., the *echogram*, and the CF images, both computed using (1) with M=3, for three different seafloor regions where marine gas seeps were identified.

### 2.3. Acoustic Backscatter from Gas-Filled Bubbles in Water

**Figure 1:**
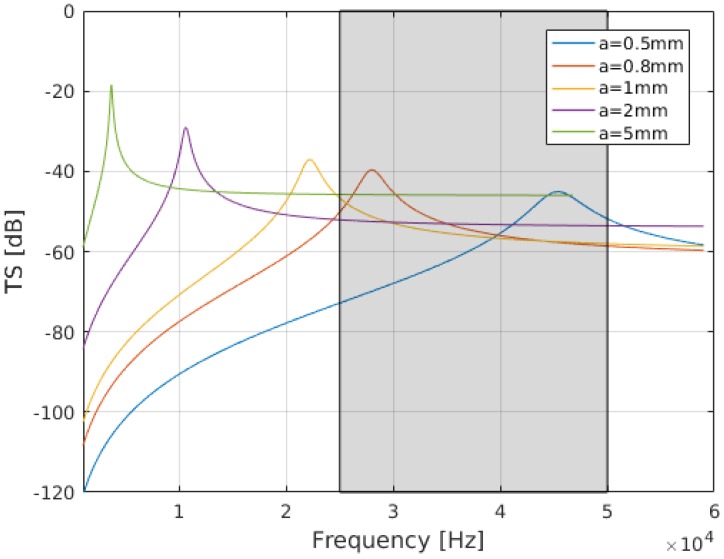
Target strength (TS) of an air-filled sphere at 500 m depth. The gray shaded area indicates the bandwidth of the EK80 split-beam transducer used in this study (25 to 50 kHz). Bubbles with a radii of approximately 0.5 mm to 0.9 mm are resonant within this frequency range. For the seeps studied here we expect predominately bubble sizes of approximately 1–5 mm radius, thus scattering above resonance.

The acoustic response of gas-filled bubbles is highly frequency dependent, with a strong peak at the resonance frequency [12,13,14,16]. The target strength (TS) from a single spherical gas bubble with radius *a* can be estimated from the total backscattering cross section $\sigma_{BS}$ as ([14,16], Chapter 3.3.3).

(2) $$\mathrm{TS}=10\log\left(\frac{\sigma_{BS}}{A_{1}}\right)=\frac{a^{2}}{{\left[{\left(\frac{f_{0}}{f}\right)}^{2}-1\right]}^{2}+\delta^{2}},$$

where *f* is the transmit frequency, $f_{0}$ the resonant frequency, *k* the acoustic wavenumber, and δ a damping term. This expression is valid for ka<1, where *k* the acoustic wavenumber. Figure 1 shows the calculated TS at 500 m water depth, for bubbles of different radii. The TS curves were calculated using (2), with δ and $f_{0}$ estimated as in [14].

The frequency range of the EK80 SBES used in this study is expected to capture the resonance peaks of bubbles with radii ranging from 0.5 mm to 0.9 mm at the relevant water depth (Figure 1). A significant fraction of the backscattered energy is likely to originate from non-resonating bubbles with radii > 0.9 mm. Extrapolating from the observations in ([41], Supplementary information), we expect bubbles with radii in the range 3–5 mm. Note that gas-filled bubbles in water are strong acoustic scatterers also in the geometric regime (above resonance).

## 3. Results

**Figure 2:**
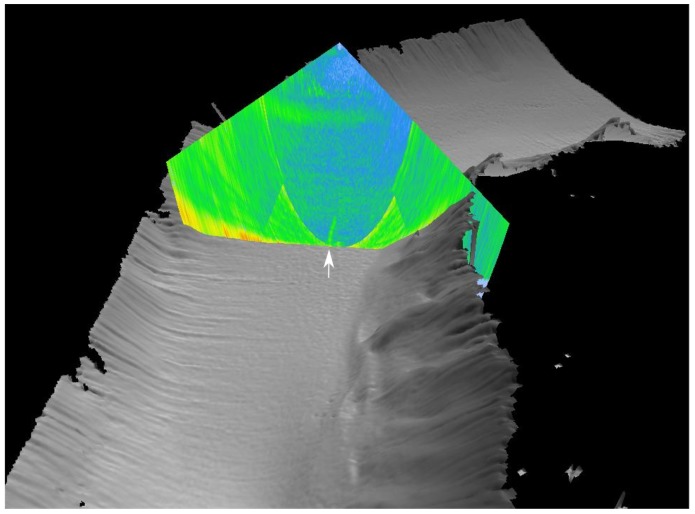
Multibeam imagery from the Hudson Canyon, acquired using the 30 kHz Kongsberg EM302 MBES mounted on the Sikuliaq research vessel. The bathymetric map of the canyon was obtained by concatenating more than one hundred pings (soundings). An acoustic backscatter image (beam fan) from a single ping shows a hydroacoustic flare indicating rising bubbles, near the center beam (indicated by the white arrow). The approximate rise height estimated from the acoustic backscatter image is around 80 m, which corresponds well with SBES observations (Figure 3a,b). This image was created using Fledermaus software.

An overview of the survey area in Hudson Canyon is shown in Figure 2. The seafloor topography map was generating using data from a Kongsberg EM302 MBES on the R/V Sikuliaq during a separate cruise in August 2014 [53]. MBES data collected on the 2016 cruise showed evidence of seeps in the area identified by Weinstein et al. [41]. For example, in Figure 2, a hydroacoustic plume is visible in the water column backscatter data from a single MBES ping, recognized as a region of relatively high backscatter and characteristic flare shape originating at the seafloor. While MBES are well suited for efficient mapping of large areas due to their wide transmit beam (here ≈120 degrees in the cross-track direction), calibrated SBES add valuable information including absolute acoustic measurements which can be used to estimate bubble flux [23,27,38,54,55].

**Figure 3:**
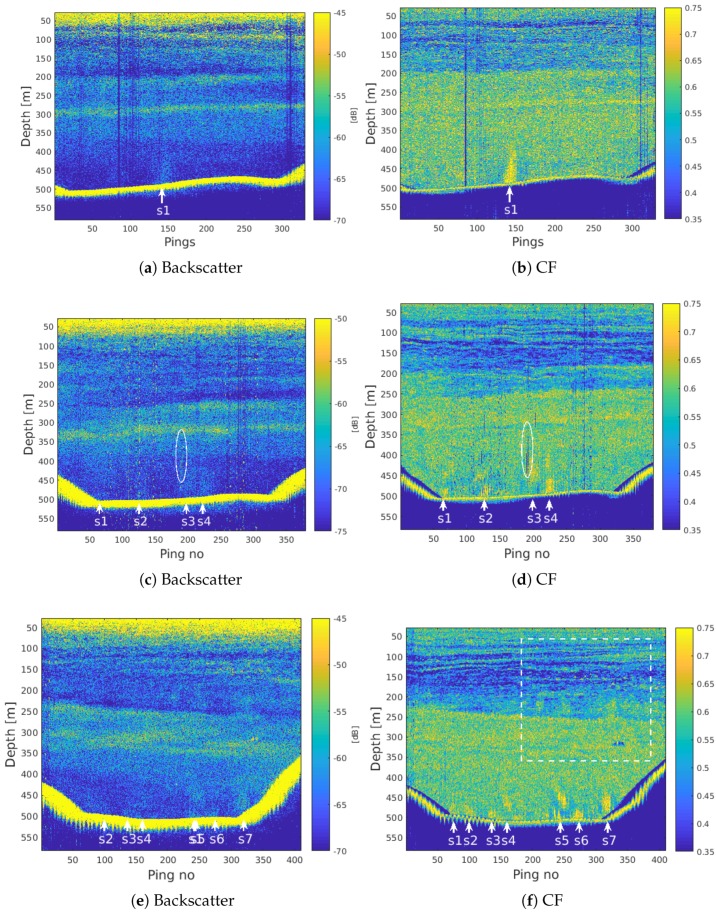
Water column backscatter images (left column), and corresponding CF images (right column), from three crosslines in the Hudson Canyon. The data were acquired using the Simrad EK80 SBES with an operating frequency centered at 37 kHz and a 25 kHz bandwidth. A vertical (depth) lowpass filter of 90 samples (corresponding to 2 m) was applied to all images for improved visualization. Notice that the scattering layers are more clearly defined in the CF image, and more details are visible. Seep positions obtained from visual inspection of the CF images are indicated by white arrows. We also observe that horizontal scattering layers are more clearly defined in the CF images, and that strong scatterers possibly related to fish or single bubbles are enhanced. The region indicated by the white rectangle in (**f**) is enlarged in Figure 4.

Figure 3 shows EK80 SBES backscatter data (left column), and CF images (right column), from three cross lines in the Hudson Canyon. All images have been lowpass filtered in the range direction using a Hanning window with a length corresponding to 2 m in the vertical direction, in order to reduce clutter and improve visualization of the hydroacoustic plumes. Manually identified seep positions are indicated by white arrows. The first crossline (Figure 3a,b) shows what is likely to be the same seep as in Figure 2. Note that the ship direction during SBES data acquisition was perpendicular to the travel path of the MBES acquisition shown in Figure 2.

In Figure 3a, a seep is located around ping 145, visible in the echogram as a region of higher backscatter than the surroundings due to scattering from bubbles in the water column. In this case, a trained operator may be able to recognize the flare indication the seep position in the echogram alone. Figure 3b shows the CF image from the same region computed using (1). The flare stands out as a confined region of high coherence (≈0.8) compared to the background at the same depth (≈0.5–0.6), and is easier to detect even for an untrained operator.

In Figure 3c, diffuse regions of relatively high backscatter strength below 400 m indicate potential seeps. However, these are not not clearly defined and may be masked by the considerable background clutter. In the CF image (Figure 3d), four distinct regions of relatively high coherence suggest seeps centered around pings 60, 125, 200, and 225. Notice that the flares appear discontinuous, which is likely to be a result of the bubble plume moving in and out of the acoustic beam. This effect can be caused by either the vessel movement, bubble trajectories affected by currents, or both.

The SBES backscatter data in Figure 3c is affected by noise, visible as vertical stripes of high intensity, and as bursts of high intensity with constant vertical spacing throughout the imaging depth. The white ellipse in Figure 3c,d indicates one of many regions affected by noise. This noise is noncoherent across the three split-beam sectors, and visible in the CF image (Figure 3d) as regions of low coherence (<0.3).

In the CF image of the third crossline (Figure 3f), seven seeps are identified through manual scrutiny. The flares related to the bubble backscatter are visible but more vaguely defined and difficult to separate in the backscatter image (Figure 3e). 3–4 of these flares appear to rise through and above the deep scattering layer (DSL), to a depth of approximately 180 m. These are qualitatively easier to track visually above the DSL in the CF image than in the backscatter image. Also notice the highly coherent pixels at around 150–200 m depth, potentially related to single fish.

**Figure 4:**
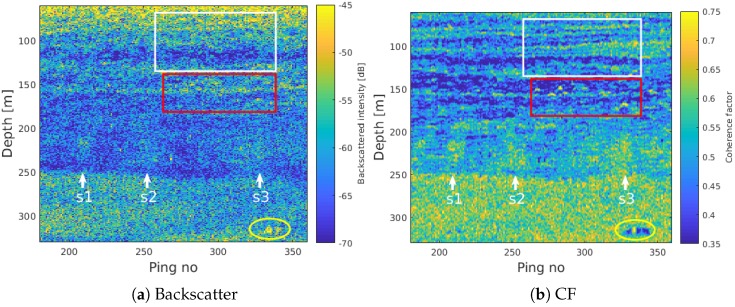
Enlargement of a region of interest from Figure 3f, showing more detail in the CF image. Single scattering objects, most likely from single fish or bubbles, can be seen in the CF image as regions of high coherence and the characteristic curved shape. Also, 3–4 flares can be tracked above the DSL, to a depth of around 200 m. A vertically extended, coherently scattering region is visible in both the echogram and in the CF image around ping 335, possibly related to a school of fish or a marine mammal.

The region indicated by the white rectangle in Figure 3f and Figure 4. Point scatterers potentially related to single fish can be recognized in the CF image by their high coherence together with the characteristic curved, nearly hyperbolic shape (red rectangle).

The yellow ellipse indicates a region with relatively high acoustic backscatter as well as coherence. This target has a vertical extent of several meters, and may be related to a school of fish or a large mammal. It is visible over more than 100 consecutive range cells when using the SBES with a 25 kHz bandwidth and a resulting range resolution of approximately *c*/2*B* = 3 cm. The CF suppresses noncoherent sidelobes, but tends to over-compensate. The regions of low coherence to either side of the highly coherent scattering object are a result of this previously documented artifact of CF processing [52]. For target detection purposes, this artifact becomes a feature enhancing its presence.

Between about 60 and 150 m depth, several thin, horizontal scattering layers are clearly defined in the CF images in Figure 3 and highlighted by the white rectangle in Figure 4b. Within the rectangle in Figure 4, eight thin layers can be separated visually. The individual layers are not separable in the SBES backscatter image (Figure 4a). These layers represent regions of relatively high backscatter intensity indicating a higher reflection coefficient than the surroundings. This may be caused by marine organisms including fish with swim bladders or plankton with air bubbles. Sharp velocity and salinity gradients may also result in similar layering effects, either on their own or because marine organisms tend to align with thermohalines. In [56,57], the authors show that there is a strong correspondence between similar acoustic layering observed using a Simrad EK80 SBES, and thermohaline stairsteps in the Arctic Ocean.

**Figure 5:**
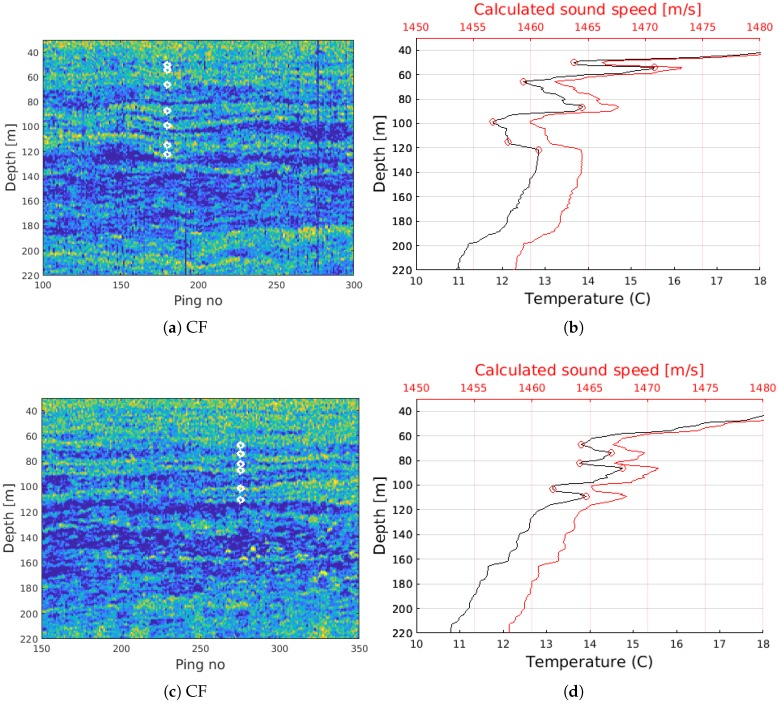
Analysis of regions of interest from Figure 3d (**a**,**b**) and Figure 3f (**c**,**d**) show good correspondence between the horizontal scattering layers observed in the CF imagery, and sharp temperature and velocity gradients derived from CTD casts. The white circles in the CF images correspond to sharp velocity gradients manually observed in the temperature depth profiles (indicated by red circles). The CTD data were acquired within 60 m of the SBES lines, and with a time offset of three hours (**a**,**b**), and 30 h (**c**,**d**). The time delay between acquisition of the SBES lines and the CTD casts allows for vertical movement and disbursement of the organisms within the scattering layer.

Analysis of a region of interest from Figure 3d,f shows good correspondence between the observed horizontal layering and sharp transitions in the measured temperature and calculated sound speed. The white circles in Figure 5a,c indicate sharp transitions in both temperature and sound speed versus depth profiles in Figure 5b,d. The temperature profiles were derived from conductivity temperature and depth (CTD) profiles acquired within 60 m of the SBES line, and the sound speed calculated using a nine-term Mackenzie equation [58]. Because there is a time offset between the line acquisition and the CTD data (approximately 3 h for Figure 5a,b, and 30 h for Figure 5c,d, a one-to-one match between the observed layers and velocity transitions is not expected. Several studies show that scattering layers consisting of different types of organisms often migrate vertically during a daily cycle [59,60,61]. Still, we observe over-all good agreement which indicates that the observed layers are related to sharp velocity gradients.

## 4. Discussion

Hydroacoustic flares related to marine gas seeps display a higher spatial coherence across the three split beam sectors of the EK80 WBT than the surrounding regions. The reason for the higher coherence is likely to be that the bubbles rising from a distinct seep location appear as a spatially focused target occupying a fraction of the EK80 footprint. Since gas-filled bubbles are highly scattering targets compared to the background, the backscatter is dominated by the bubbles which act as strong, relatively coherent scatterers. As a result, the flares appear enhanced in the CF image, potentially simplifying the considerable manual labor involved with identifying seeps in large data sets. This is especially relevant in deep waters, for small seeps, when background noise levels are considerable, or when rising bubbles are masked by the deep scattering layer (DSL). While the hydroacoustic plumes observed in this study appeared focused, i.e., originating from a confined spatial region, more distributed seepage may also occur for instance along a fault line. Small, distributed seeps are more difficult to detect in both MBES and SBES imagery, and the spatial coherence is not expected to enhance their presence.

It is worth noting that the acoustic anomalies related to gas bubbles could also be associated with other acoustic scatterers, including fish [54,55,62]. While fish with swim bladders display target strengths which are comparable to that of gas bubbles with similar radii, hydroacoustic flares can often be identified due to their characteristic shape as they rise through the water column. It is likely that the observed acoustic anomalies in this study are associated with gas bubbles based on the shape and rise height of the acoustic anomalies in the SBES data, as well as analysis of MBES water column data from the same region. The assumption is strengthened further by other evidence from a previous study in the same area [41], including geophysical evidence of gas below the seabed, and elevated aqueous CH4 concentrations at depths corresponding to the acoustic anomalies identified in our present manuscript as bubble trains. This is also the same area where gas seeps were previously identified Skarke et al. [42].

Hydroacoustic plumes sometimes appear discontinuous in the echogram, with parts of the plume missing. This is a result of the limited beamwidth of the split-beam echo sounder, and is discussed and illustrated in [23]. When the vessel does not pass directly above the seep or within the beamwidth (here ≈10 degrees), the bubbles are not insonified and therefore not included in the echogram. Similar affects occur in the multibeam imagery, caused by the narrow along-track receive beam (typically between 0.5 and 2 degrees). This feature/artifact may be emphasized in the SBES aperture coherence image, since the spatial coherence of a plume reaches its maximum when the bubbles are located in the center of the beam. This may also result in an apparent improvement in angular resolution since the bubbles will only display coherence over a single or a few pings, while the echogram will display an increased target strength over a wider range of pings. This effect is visible for the target within the yellow ellipse in Figure 4, which spans multiple (≈5) pings in the backscatter image (Figure 4a) but is more focused in the CF image (≈3 pings) (Figure 4b). While this may be an advantage when identifying multiple closely spaced targets in deep water, the erroneously low CF values for off-axis targets may introduce errors in other applications. For instance, the ability to differentiate between single rising bubbles and fish may be reduced since this involves analyzing the acoustic response over a wide range of angles, and some of these will be suppressed in the CF image.

CF processing may be beneficial to other application within oceanography. Experience from research addressing the CF in other domains such as medical ultrasound imaging suggests that CF processing is an efficient tool for enhancing point targets in noisy environments [63]. Building on these findings, it is reasonable to assume that the CF will be successful in enhancing the presence of fish in the water column. We also observe that horizontal scattering layers caused either by an accumulation of marine organisms, sharp velocity and salinity gradients, or a combination of the two, are more clearly defined in the CF image than in the acoustic backscatter image. Closely spaced individual layers are separable in the CF image while this is not the case in the corresponding acoustic backscatter image.

Without knowing the physical origin of these layers in this case, we cannot fully explain why they display a relatively high aperture coherence. Possible explanations are that the scattering layer is smooth relative to the scale of measurement, resulting in a high reflection coefficient and coherent scattering ([64], Chapter 3), or that the layer contains a sparse distribution of strongly scattering single targets such as small fish which result in a coherently scattered wavefield.

The CF image and the acoustic backscatter image from a calibrated SBES offer complementary information, and it may be useful to analyze them together for a better understanding of the marine environment. The CF represents an alternative way to process split-beam echo sounder data in which bubbles and other highly coherent scatterers are enhanced. The visual enhancement of these features may lead to a better understanding of the vertical and horizontal extent of these features, and their evolution over time. In order to relate these observations to absolute quantities including bubbles size distributions and seep flux, one would revert to the calibrated water column backscatter data (*I* in (1)).

Finally, automatic algorithms for gas seep detection from acoustic imagery is a highly relevant topic for current and future research. Recent advances in statistical image analysis including machine learning algorithms show promising results in other areas. The spatial coherence measured across the receiver may add valuable information making automatic detection more robust.

## 5. Conclusions

Mapping naturally occurring marine gas seeps is an overwhelming task due to the large extent of marine environments where seepage may occur. In addition, the data processing and manual interpretation involved is time consuming and highly labor-intensive. We demonstrate that the spatial coherence of the wavefield measured across the split-beam aperture, quantified by the CF, may be used to enhance hydracoustic plumes in acoustic imagery and simplify the considerable manual labor associated with mapping marine gas seeps. We also observe that other physical oceanography features including horizontal scattering layers are more clearly defined and resolved in the CF images. As accumulations of plankton and other marine organisms tend to align with thermohalines and internal waves, CF processing may be a helpful tool in tracking these features and mapping their vertical and horizontal extent.

## Supplementary Materials

The Supplementary Materials are available online: https://zenodo.org/record/1298394.
